# Supplementary material for: Optical Sensing of Humidity Using Polymer Top-Covered Bragg Stacks and Polymer/Metal Thin Film Structures
Source: Nanomaterials (Basel). 2019 Jun 10;9(6):875. doi: 10.3390/nano9060875 (PMC6630599; doi:10.3390/nano9060875)
Supplement: Supplementary file 1 [file nanomaterials-09-00875-s001.pdf]

nanomaterials

Supplementary Material

Optical Sensing of Humidity Using Polymer Top-Covered Bragg Stacks and Polymer/Metal Thin Film Structures

Katerina Lazarova [1,*], Darinka Christova [2], Rosen Georgiev [1], Biliana Georgieva [1] and Tsvetanka Babeva [1,*]

1 Institute of Optical Materials and Technologies ''Acad. J. Malinowski'', Bulgarian Academy of Sciences, Akad. G. Bonchev str., bl. 109, 1113 Sofia, Bulgaria; rgeorgiev@iomt.bas.bg (R.G.); biliana@iomt.bas.bg (B.G.)
2 Institute of Polymers, Bulgarian Academy of Sciences, Akad. G. Bonchev Str., bl. 103-A, 1113 Sofia, Bulgaria; dchristo@polymer.bas.bg
* Correspondence: klazarova@iomt.bas.bg (K.L.); babeva@iomt.bas.bg (T.B.); Tel.: +359-02-979-3526 (K.L.); +359-02-872-0073 (T.B.)

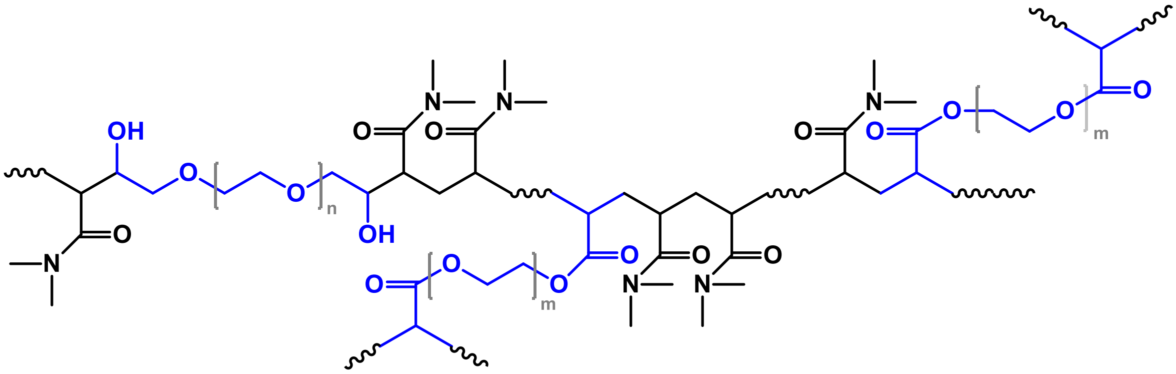

Figure S1. Schematic presentation of the structures of multiblock copolymer with branched macromolecular architecture comprising poly(N,N-dimethyl acrylamide) (PDMA) and poly(ethylene oxide) (PEO) segments.

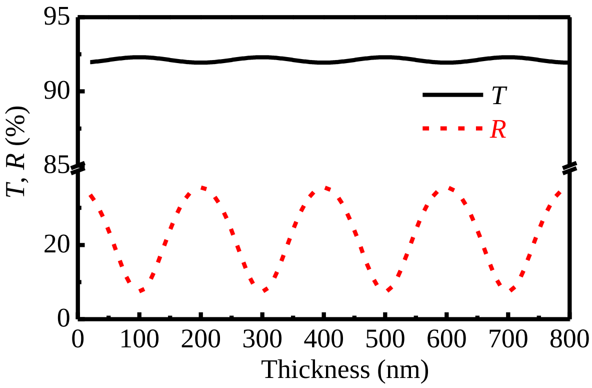

Figure S2. Calculated transmittance and reflectance at wavelength of 600 nm of transparent thin film with refractive index of 1.5 deposited on glass substrate as function of thickness.

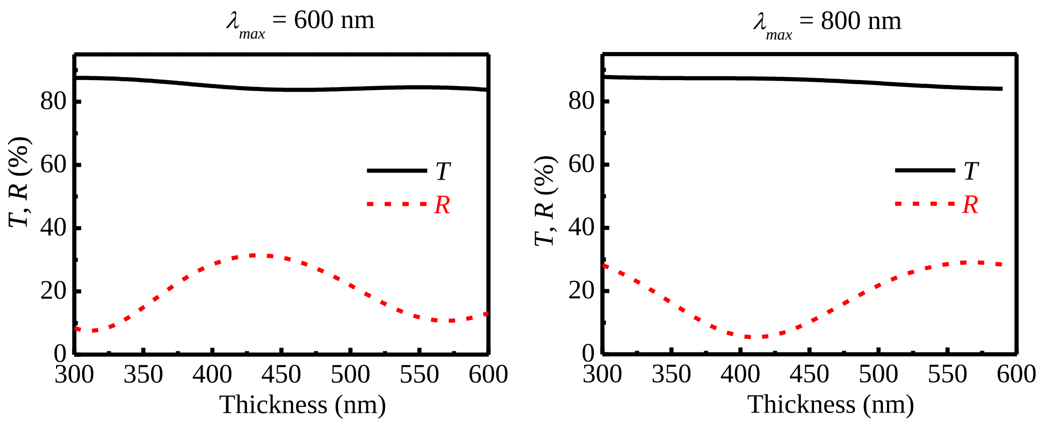

Figure S3. Calculated transmittance and reflectance at wavelengths of 600 nm (Left) and 800 nm (Right) of transparent thin film with refractive index of 1.5 deposited on glass substrate and silicon wafer as function of thickness.

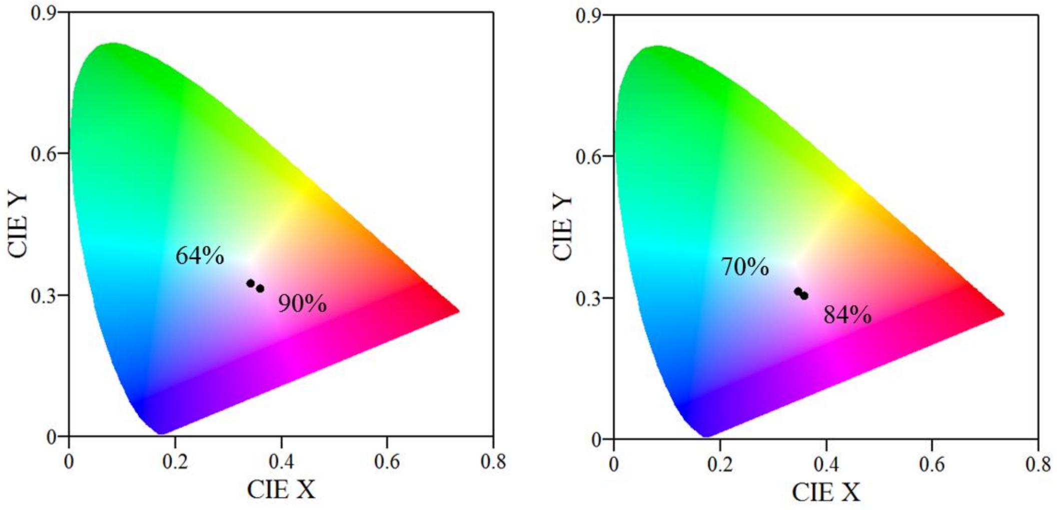

Figure S4. CIE color coordinates of 5-layers Bragg stack top-covered with 300 nm polymer film exposed to denoted levels of relative humidity.
